# Supplementary material for: Complexome profiling of the Chlamydomonas psb28 mutant reveals TEF5 as an early PSII assembly factor
Source: Plant Cell. 2025 Mar 17;37(6):koaf055. doi: 10.1093/plcell/koaf055 (PMC12164590; doi:10.1093/plcell/koaf055)
Supplement: koaf055_Supplementary_Data [file koaf055_supplementary_data.zip › psb28_interactive.zip!/plots/intensity/qualityPlots/peptides/GPX1_Cre02.g078300.t1.1.html]

### Cre02.g078300.t1.1

Trivial name: GPX1  
SeqCoverage [median]: 71.60  
Mass: 7.29 kDa  
  
 

| Sequence | MissedCleavage | Mass [Da] | wtIntensity | muIntensity | Score |
| --- | --- | --- | --- | --- | --- |
| FLVRPDGTVFGR | 1 | 1362.74 | 4148.72 | 20390.65 | 74.76 |
| VAAGDTSDIGWNFGK | 0 | 1536.72 | 342.28 | 2795.93 | 78.65 |
| YAPTTGPLSLEK | 0 | 1275.67 | 1237.99 | 3580.35 | 77.38 |
| YIVELINSR | 0 | 1105.61 | 1216.75 | 4585.56 | 89.36 |
|  |  |  | 6945.7 | 31352.5 |  |
